# Comparative Safety Analysis of Avastin and Bevacizumab Biosimilars Based on Food and Drug Administration Adverse Event Reporting System

**DOI:** 10.1111/bcpt.70099

**Published:** 2025-08-27

**Authors:** Xiaoyu Zhang, Yupeng Zhang, Xinghang Tang, Li Chen

**Affiliations:** ^1^ Department of Equipment West China Hospital, Sichuan University Chengdu China; ^2^ Evidence‐Based Pharmacy Center West China Second Hospital, Sichuan University Chengdu China; ^3^ Key Laboratory of Birth Defects and Related Diseases of Women and Children (Sichuan University), Ministry of Education Chengdu China; ^4^ West China School of Pharmacy Sichuan University Chengdu China; ^5^ Department of Pharmacy West China Second Hospital, Sichuan University Chengdu China

**Keywords:** bevacizumab, biosimilars, FAERS, safety, signal comparison

## Abstract

**Objective:**

This study aimed to compare adverse event (AE) profiles between Avastin and bevacizumab biosimilars to support clinical decision‐making, given the limited availability of real‐world data.

**Methods:**

A disproportionality analysis was conducted using the FDA Adverse Event Reporting System (FAERS) to identify and compare AE signals. Signals were evaluated at the system organ classes (SOCs) and preferred term (PT) levels, focusing on the Top 20 PTs by report number, key SOCs and outcomes.

**Results:**

Injury, poisoning and procedural complications and general disorders and administration site conditions were the most frequent SOCs in both groups. Common label‐listed AEs, including hypertension, proteinuria and thrombocytopenia, were frequently reported. Shared risks also included gastrointestinal perforation/ulceration and thromboembolism. Avastin was more associated with red blood cell disorders and ureteric disorders and bladder and bladder‐neck disorders, while biosimilars were linked to a broader range of high‐level group terms in gastrointestinal disorders and generated more renal and urinary signals.

**Conclusion:**

Hypertension, proteinuria, thrombocytopenia, gastrointestinal perforation and thromboembolism remain key concerns. Clinicians should monitor renal and urinary function when administering Avastin. Immune‐induced renal disorders associated with biosimilars highlight the importance of assessing the treatment rationale in patients with chronic kidney disease, autoimmune disorders or other comorbid conditions.

## Summary

This study used real‐world safety data to compare side effects of Avastin and its biosimilar versions. We found that both types of drugs often caused high blood pressure, protein in the urine and low platelet counts. However, biosimilars were linked to more kidney and urinary problems, while Avastin had more red blood cell and bladder‐related side effects. These findings suggest doctors should closely monitor kidney function and consider a patient's other health conditions when choosing between Avastin and its biosimilars.

**Abbreviations**
DE: death
DS: disability
HO: hospitalization‐initial or prolonged
LT: life‐threatening
OT: other serious (important medical event)
RI: required intervention to prevent permanent impairment or damage

## 1 Introduction

Vascular Endothelial Growth Factors (VEGFs)and Vascular Endothelial Growth Factor Receptor (VEGFR) have been shown to play a critical role in blood vessel development, angiogenesis, endothelial cell function and the regulation of vascular tone [1, 2]. Bevacizumab, the first monoclonal antibody used in clinical practice as an anti‐VEGFR agent, has been widely applied in the treatment of colon cancer, non‐small cell lung cancer and other tumours since 2004 [3]. To date, the Food and Drug Administration (FDA) has approved six biosimilars from various manufacturers [3]. Substantial evidence supports the efficiency of both bevacizumab and its biosimilars [4, 5, 6, 7, 8]. Common adverse reactions including hypertension, bleeding, venous and arterial thromboembolic events, proteinuria, wound healing complications and gastrointestinal perforation have been reported in previous studies [9, 10]. However, systematic reviews assessing safety differences between bevacizumab and its biosimilars, particularly in patients with complex comorbidities, are lacking.

## 2 Materials and Methods

### 2.1 Data Sources and Processing

This retrospective pharmacovigilance study analysed data from the FDA Adverse Event Reporting System (FAERS), encompassing reports submitted between the first quarter of 2020 and the fourth quarter of 2024 that contained anonymized adverse event (AE) data accessible to investigators. We extracted data from key tables, including demographic and administrative information (DEMO), drug information (DRUG), adverse events coded with preferred terms (REAC) and patient outcomes (OUTC). Reporters included healthcare professionals (e.g., physicians, pharmacists and other health practitioners) and non‐healthcare reporters (e.g., consumers and lawyers). The study adhered to the Basic and Clinical Pharmacology and Toxicology policy for experimental and clinical studies [11] and followed the Reporting of studies Conducted using Observational Routinely‐collected Data (RECORD) guidelines [12].

Invalid or withdrawn records were first removed by consulting the DELETED file in FAERS. The remaining reports were then deduplicated in line with the FDA's two‐step procedure. When multiple reports shared the same ‘caseid’, reports with the most recent submission date (‘fda_dt’) were retained. When multiple records had the same case ID (‘caseid’) and submission date (‘fda_dt’), we retained the report with the highest ‘primaryid’ value. For drug identification, reports were restricted to those in which the ‘drugname’ corresponded to either the reference biologic Avastin or any of the following FDA‐approved bevacizumab biosimilars: Alymsys, Avzivi, Mvasi, Vegzelma and Zirabev. Additionally, the active ingredient (‘prod_ai’) was required to be ‘bevacizumab’ and the role code (‘role_cod’) was limited to ‘PS’ (primary suspect). Considering the insufficient number of reports and the potentially large reporting bias, the study did not include Jobevne, which was launched on April 9 2025. Reports referring to the counterfeit product Altuzan were excluded.

AEs associated with bevacizumab were systematically classified using the Medical Dictionary for Regulatory Activities (MedDRA, Version 27.1). Data analysis was performed using Microsoft Excel 2016. This study utilized publicly available anonymized data and did not involve any personal privacy information. Accordingly, the study fell under the exemption criteria outlined in the Declaration of Helsinki: Ethical Principles for Medical Research Involving Human Subjects [13].

### 2.2 Data Mining

Two widely recognized pharmacovigilance metrics: reporting odds ratio (ROR) and proportional reporting ratio (PRR) were employed to assess the safety profile of Avastin and biosimilars. Specific standards and details were shown in Tables 1 and 2.

**TABLE 1:** Fourfold table of measures of disproportionality.

Drug category	Adverse event of interest	All other adverse events	Total
Drug of interest	a	b	a + b
All other drugs in FAERS	c	d	c + d
Total	a + c	b + d	*N* = a + b + c + d

**TABLE 2:** Formulas and thresholds of the ROR method and the MHRA method.

Method	Formula	Threshold value
ROR	ROR = ad/bc SE (lnROR) = $\sqrt{\left(\frac{1}{a}+\frac{1}{b}+\frac{1}{c}+\frac{1}{d}\right)}$ $95\%\mathrm{CI}=e^{\ln\left(\mathrm{ROR}\right)\pm 1.96\sqrt{\frac{1}{a}+\frac{1}{b}+\frac{1}{c}+\frac{1}{d}}}$	A signal is detected, if a ≥ 3, and the lower limit of the 95% (ROR)> 1
MHRA	PRR = $\frac{a/\left(a+b\right)}{c/\left(c+d\right)}$ $\chi^{2}=\frac{{\left(a\mathrm{d}-\mathrm{bc}\right)}^{2}*N}{\left(a+b\right)\left(c+d\right)\left(b+d\right)\left(a+c\right)}$	A signal is detected, if a ≥ 3 and PRR ≥ 2, and χ2 ≥ 4

Abbreviations: MHRA, Medicines and Healthcare products Regulatory Agency; PRR, proportional reporting ratio; ROR, reporting odds ratio.

### 2.3 Data Presentation

Descriptive analyses were conducted to summarize demographic characteristics, including gender, age, country of origin and the occupations of reporters. Signal detection contained levels of system organ class (SOC), high‐level group term (HLGT) and preferred term (PT) to evaluate differences between Avastin and biosimilars. The Top 20 PTs ranked by report number were identified and visualized to highlight the most commonly reported events. In addition, the Top 20 PTs within four key SOC categories, including gastrointestinal, vascular and lymphatic, hematologic and renal and urinary systems, were identified based on ROR values to assess drug‐specific safety signals. Given the substantial variability in raw frequency counts and ROR values, natural logarithm (ln) transformation was applied to improve data normalization and interpretability. Data visualization was performed using Python 3.13 and GraphPad Prism 10.1.2.

## 3 Results

### 3.1 Descriptive Analysis

**FIGURE 1:**
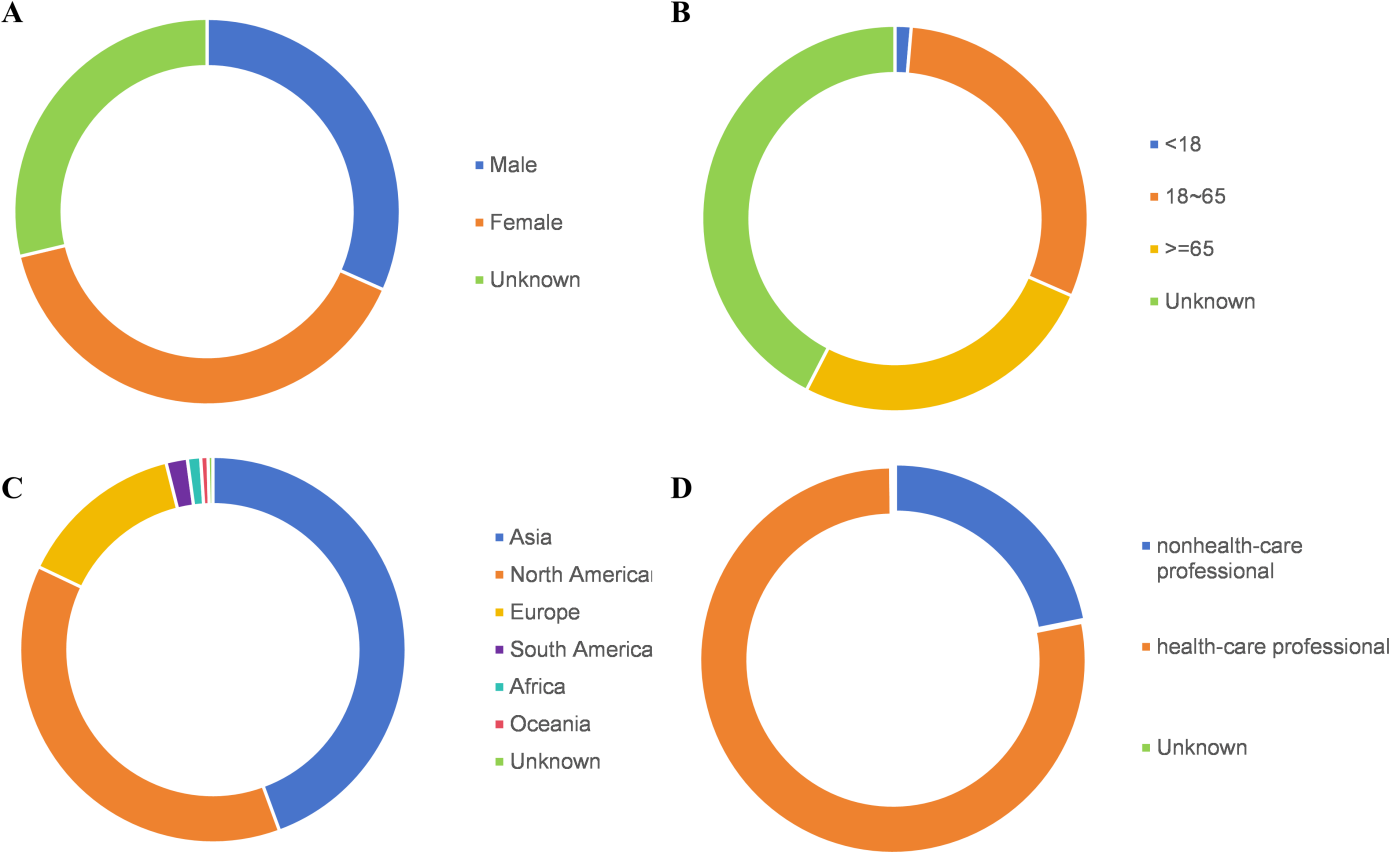
Demographic and reporter characteristics of adverse event reports associated with Avastin in FAERS (2020Q1–2024Q4): (A). gender distribution; (B). age groups; (C). geographical regions; (D). reporter occupation.

**FIGURE 2:**
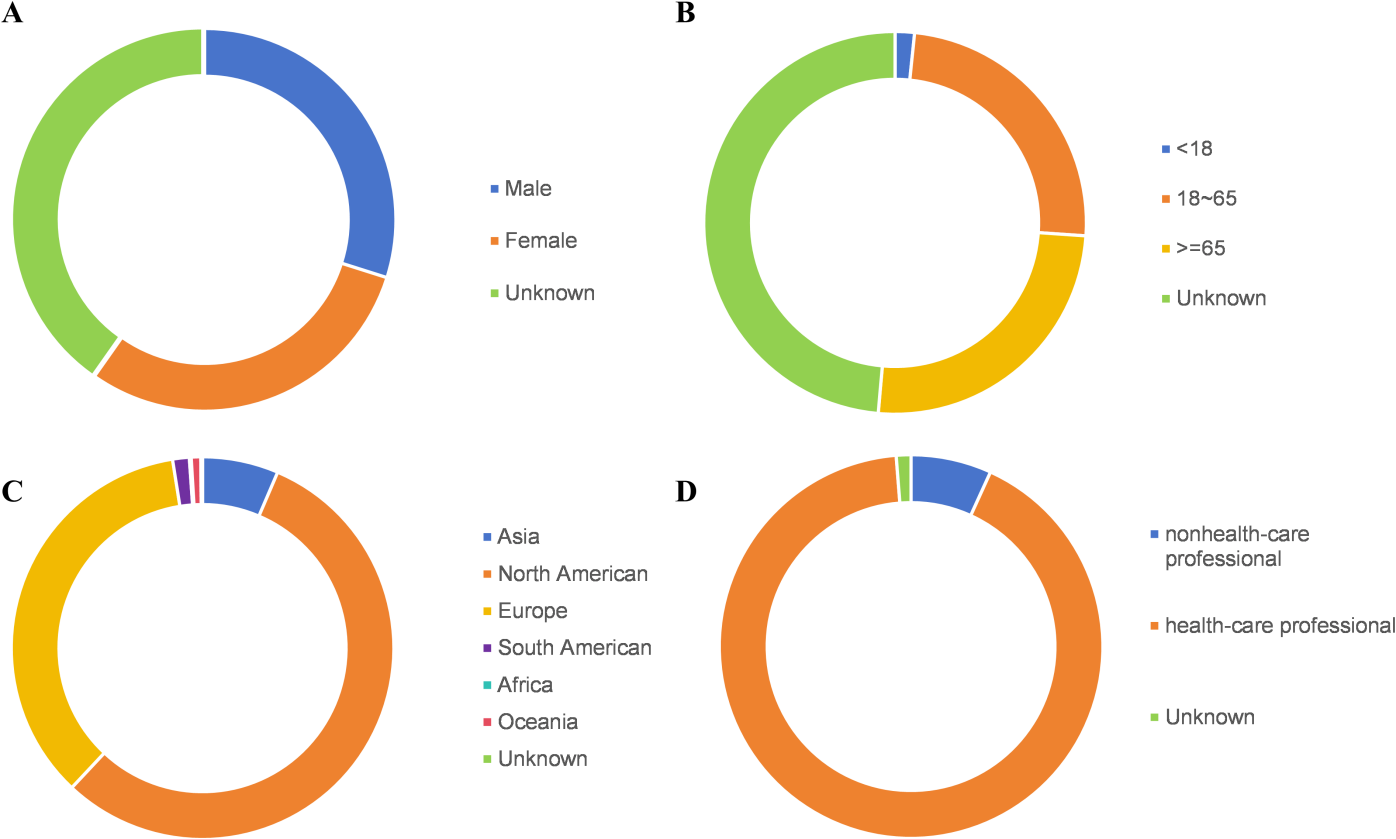
Demographic and reporter characteristics of adverse event reports Associated with bevacizumab biosimilars in FAERS (2020Q1–2024Q4): (A). gender distribution; (B). age groups; (C). geographical regions; (D). reporter occupation.

From the first quarter of 2020 to the fourth quarter of 2024, the FAERS database documented 7 434 049 individual case reports including 14 422 cases associated with Avastin and 10 222 cases involving its five biosimilars. The characteristics of these cases are presented in Figures 1 and 2. Among Avastin‐associated cases, 31.63% were male and 39.63% female. In the biosimilar group, the proportions of male and female patients were nearly equal, at 29.89% and 29.88%, respectively. Most reports pertained to adult patients, while paediatric cases were relatively infrequent. Cautiously, gender and age were unknown or missing to a large degree for Avastin and biosimilars. Geographically, most reports originated from Asia, North America and Europe. Both drug categories were predominantly reported by healthcare professionals, accounting for 77.76% of reports for Avastin and 99.96% for biosimilars.

### 3.2 Signal Detection

#### 3.2.1 System Organ Class Comparison

**FIGURE 3:**
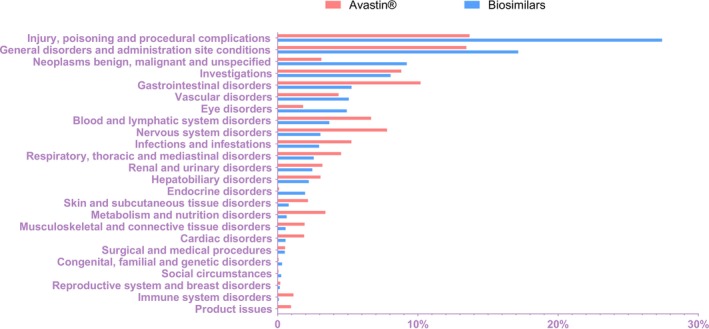
Distribution of adverse events at the system organ class level for Avastin and its biosimilars.

The signal detection results at SOC level for Avastin and its biosimilars are shown in Figure 3. For both drug categories, the top two were injury, poisoning and procedural complications and general disorders and administration site conditions. Beyond these leading categories, AEs related to Avastin were primarily concentrated in gastrointestinal disorders, investigations, nervous system disorders, blood and lymphatic system disorders and infections and infestations. In contrast, biosimilar‐associated reports, apart from the aforementioned SOCs, most frequently involved neoplasms benign, malignant and unspecified investigations gastrointestinal disorders and vascular disorders. Notably, both Avastin and its FDA‐approved biosimilars indicated similar proportional composition in investigations, vascular disorders, renal and urinary disorders and hepatobiliary disorders.

#### 3.2.2 PT Analysis and Comparison of Adverse Reaction Frequencies

According to the Top 20 PTs ranked by report number (Table 3), off label use was the most frequently reported PT both for Avastin and its biosimilars. Several common AEs listed in the product labels, including proteinuria, hypertension, thrombocytopenia and anaemia, were all included among the Top 20 PTs for them. They were associated with AEs spanning 10 SOC categories. Reports related to Avastin were predominantly focused on blood and lymphatic system disorders, including white blood cell disorders, platelet disorders and anaemias nonhaemolytic and marrow depression. In contrast, biosimilar‐associated reports were predominantly related to general disorders, administration site conditions and injury, poisoning and procedural complications. Notably, product storage error appeared exclusively among the Top 20 PTs for biosimilars.

**TABLE 3:** Top 20 PTs for Avastin and its biosimilars ranked by report number.

PTs	Avastin report number	Avastin ROR	Biosimilars report number	Biosimilars ROR
Off label use	3634	5.34	2906	6.28
Death	—	—	1132	2.81
Myelosuppression	1051	28.79	—	—
Hypertension^a^	803	6.14	507	5.41
Intentional product use issue	654	6.52	440	6.14
Product storage error	—	—	399	5.53
Drug effective for unapproved indication	—	—	324	11.55
Circumstance or information capable of leading to medication error	—	—	319	13.81
Disease progression	653	7.57	270	4.29
Anaemia	530	4.73	249	3.07
Therapy partial responder	—	—	247	20.75
Proteinuria^a^	443	35.16	237	25.52
Neutropenia	402	3.5	—	—
Decreased appetite	391	2.46	—	—
Platelet count decreased^a^	386	5.13	172	3.17
Blood pressure increased^a^	374	3.56	222	2.95
Endophthalmitis	366	105.2	—	—
Thrombocytopenia^a^	353	4.9	203	3.94
White blood cell count decreased^a^	311	3.82	—	—
Blindness	275	10.27	—	—
Neutrophil count decreased	264	8.15	—	—
Renal impairment	227	3.7	—	—
Adverse event	—	—	222	6.34
Hepatocellular carcinoma^a^	—	—	219	84.72
Haemorrhage^a^	215	3.29	—	—
Epistaxis^a^	215	4.9	—	—
Neuropathy peripheral	212	2.99	—	—
Drug ineffective for unapproved indication	—	—	182	5.43
Neoplasm progression	—	—	164	6.56
Hypothyroidism	—	—	147	9.21
Colorectal cancer metastatic	—	—	136	146.12

*Note:* PTs are ranked by report number in Avastin cases; those not reported for Avastin are ranked by report number in biosimilars.

^a^ Adverse events listed in the official prescribing information for both Avastin and its biosimilars.

### 3.3 Signal Comparison Analysis

**FIGURE 4:**
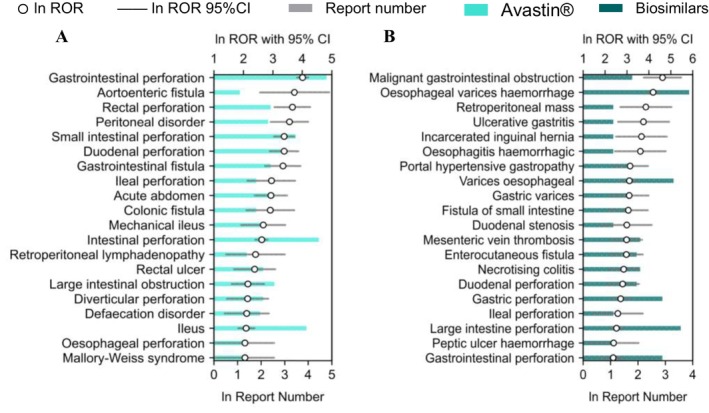
Top 20 preferred terms related to gastrointestinal disorders associated with Avastin and its biosimilars, ranked by reporting odds ratio: (A). Avastin; (B). biosimilars.

**FIGURE 5:**
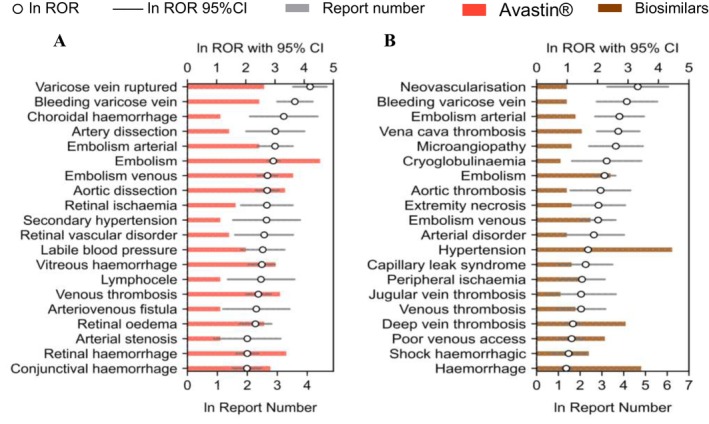
Top 20 preferred terms related to vascular and lymphatic disorders associated with Avastin and its biosimilars, ranked by reporting odds ratio: (A). Avastin; (B). biosimilars.

**FIGURE 6:**
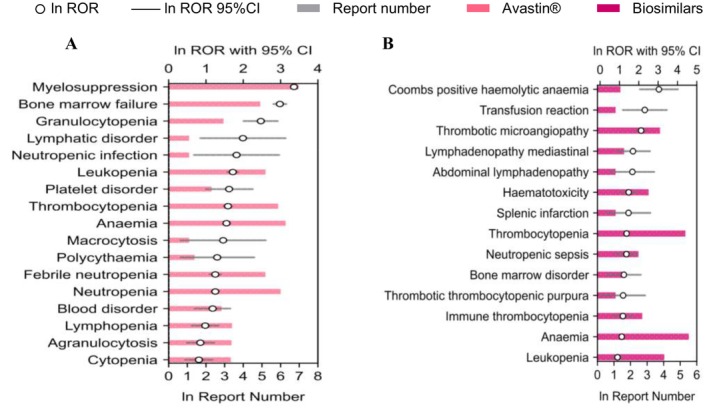
Top 20 preferred terms related to hematologic and lymphatic disorders associated with Avastin and its biosimilars, ranked by reporting odds ratio: (A). Avastin; (B). biosimilars.

**FIGURE 7:**
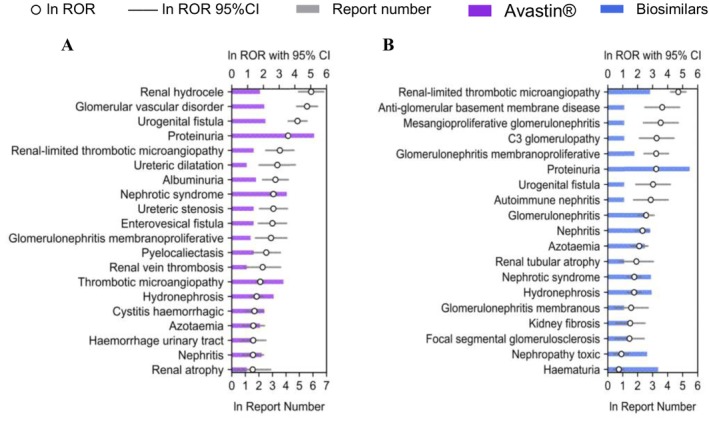
Top 20 preferred terms related to renal and urinary disorders associated with Avastin and its biosimilars, ranked by reporting odds ratio: (A). Avastin; (B). biosimilars.

Based on these findings, we further compared Avastin and its biosimilars across four clinically significant SOCs: renal and urinary disorders, gastrointestinal disorders, vascular and lymphatic disorders and hematologic disorders. Differences were quantified through the number of reports and RORs, as shown in Figures 4, 5, 6, 7.

#### 3.3.1 Gastrointestinal Toxicity

In gastrointestinal disorders, Avastin and its biosimilars covered 14 and 12 types of HLGTs, respectively. Both drug types showed the highest number of PTs within the HLGT of gastrointestinal ulceration and perforation. In this HLGT category, Avastin exhibited relatively high ROR values for gastrointestinal perforation, rectal perforation, small intestinal perforation, duodenal perforation and ileal perforation. Particularly, within the HLGT of hepatobiliary disorders, biosimilars covered the highest number of PTs among the six unique categories, with the top three PTs ordered by ROR being oesophageal varices, portal hypertensive gastropathy and varices oesophageal.

#### 3.3.2 Vascular and Lymphatic Toxicity

The characteristics of vascular and lymphatic related AEs at the level of HLGT were generally similar between Avastin and its biosimilars. Both drug types showed a concentration of reports related to embolism and thrombosis but Avastin involved a broader range of PT categories in this HLGT, covering 12 categories compared to fewer in biosimilars. Both showed relatively high ROR values for embolism arterial. In terms of report frequency, the top three PTs were hypertension, haemorrhage and deep vein thrombosis for both Avastin and biosimilars. Moreover, varicose vein ruptured showed a significant safety signal specifically associated with Avastin.

#### 3.3.3 Hematologic Toxicity

In blood and lymphatic system disorders, Avastin covers 8 HLGTs, while biosimilars cover 7 HLGTs. A total of 17 PTs related to Avastin were mainly concentrated in white blood cell disorders. Avastin‐related red blood cell disorders comprised macrocytosis and polycythaemia, for which no corresponding signal was observed in biosimilars, suggesting these may be specific AEs associated with Avastin. In contrast, biosimilars were linked to 14 PTs related to hematologic toxicity.

#### 3.3.4 Renal and Urinary Toxicity

Avastin caused 22 category signals involving 6 different HLGTs and focused on renal disorders (excl nephropathies). Biosimilars generated 19 signals in renal and urinary disorders, which were distributed across 4 different HLGTs. In renal disorders (excl nephropathies), renal hydrocele and glomerular vascular disorders correlated tremendously with Avastin. Additionally, Avastin was associated with ureteric disorders, as well as bladder and bladder‐neck disorders (excl calculi) but biosimilars generated no signals, including ureteric dilatation, ureteric stenosis, enterovesical fistula and cystitis haemorrhagic. However, biosimilars generated signals in nephropathies and covered 12 types of PTs, while Avastin just covered 3 types. For example, anti‐glomerular basement membrane disease, mesangioproliferative glomerulonephritis and C3 glomerulopathy were significantly related to biosimilars.

### 3.4 Outcome Proportion due to Bevacizumab‐Associated Disorders

**FIGURE 8:**
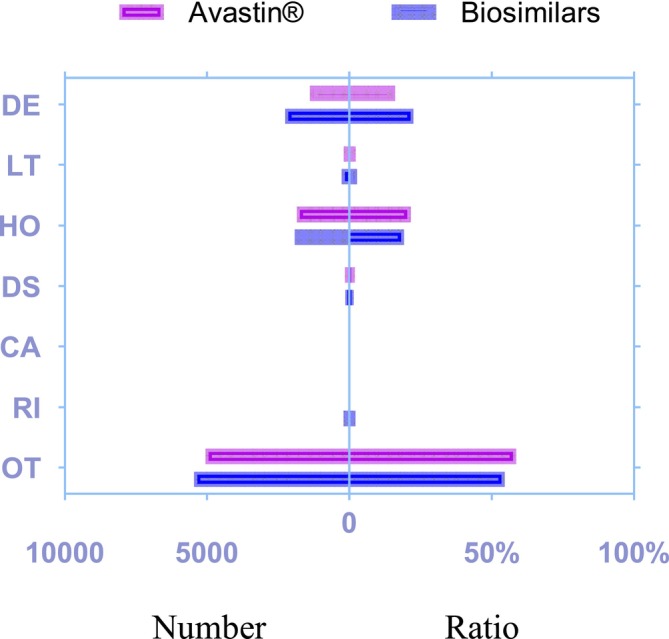
Distribution of adverse event outcomes for Avastin and its biosimilars.

The statistical analysis of AE outcomes for bevacizumab is presented in Figure 8, where outcomes are ordered from top to bottom in decreasing severity. More AE reports for both Avastin and its biosimilars were observed in the outcome categories of death, hospitalization‐initial or prolonged and other serious outcomes, while fewer reports were found in life‐threatening, disability and required intervention to prevent permanent impairment or damage. Compared with biosimilars, Avastin showed a lower proportion of death outcomes but a higher proportion of other serious outcomes among reported cases.

## 4 Discussion

### 4.1 Difference in Safety Between Avastin and Biosimilars

#### 4.1.1 Difference in Manufacture Process

Compared to chemical drugs, biological products are characterized by complex structures and unique immunological properties [14, 15]. Due to regulatory and patent restrictions, manufacturers of biosimilars must establish independent production systems, including the selection of cell lines, as well as the optimization of culture conditions and purification parameters [16].

By employing the ‘orthogonal verification’ strategy, which integrates multiple methodologies, such as mass spectrometry, spectroscopy, crystallography and thermodynamic analysis, a comprehensive analysis can be conducted to assess the high degree of similarity between the primary, secondary and tertiary structures of the biosimilar and the original drug [17, 18, 19]. Although current detection techniques are considered valid by EMA, numerous studies have shown that changes, such as glycosylation and aggregate generation, both of which are difficult to control, can influence the properties of biological products [20, 21, 22]. Additionally, temperature fluctuations can affect the production of specific antibodies in Chinese hamster ovary cells [23]. Freezing and thawing may also impact the stability of biological products [24]. The observed differences in SOC‐level AE distributions between Avastin and its biosimilars may be related to these factors.

#### 4.1.2 Difference in Inactive Ingredients

Excipients in formulation are usually considered to have significant and complicated impacts on product quality and safety. We found immunization‐related disorders include anti‐glomerular basement membrane disease, mesangioproliferative glomerulonephritis and complement three glomerulopathy had significantly related to biosimilars. Bevacizumab injections typically use polysorbate (PS) 20 as a stabilizer, while Zirabev uses PS80 stabilizing protein. Most commercially available protein preparations use PS80 and PS20 as surfactants to stabilize proteins. However, the degradation caused by the hydrolysis and oxidation of side chains may affect the stability of biopharmaceutical preparations [25]. Research showed excipient selection, buffer chemistry and packaging materials could influence the degradation of PS, whether PS20 or PS80 [26, 27]. It can be seen from the FDA's approval documents that the five bevacizumab biosimilars are slightly different from Avastin in terms of quality attributes, such as glycosylation, acidic or basic variants, although these are considered to be minor clinically inactive component variations. Additionally, studies suggest sugars including trehalose as a component for bevacizumab usually as a stabilizer in formulation also influence stability [28]. Thus, much further evidence needs to validate the impact on excipient.

#### 4.1.3 Quality Control During Transportation and Storage

Biological products require stringent transportation and storage conditions to maintain their stability. In our study, product storage error was identified as one of the Top 20 PTs ranked by report number for biosimilars, highlighting the importance of controlling factors that may compromise product properties. Previous studies have emphasized that increasing supply chain flexibility can help balance supply and demand effectively [16]. For example, bevacizumab must be stored at 2°C–8°C (36°F–46°F) in the original carton and protected from light until administration [3]. Furthermore, studies have verified that different types of light exposure can lead to varying degrees of product degradation [29]. Therefore, both pharmaceutical manufacturing enterprises and healthcare providers should implement robust storage and handling protocols to ensure the quality and efficacy of biological drugs.

### 4.2 Clinical Significance and Application

Based on our analyses, bevacizumab biologic and biosimilars generated signals in proteinuria, hypertension, thrombocytopenia and anaemia similarly in the results of Top 20 PTs. Meanwhile, they were prone to gastrointestinal ulceration and perforation, embolism and thrombosis. These AEs mentioned in labels may link to the pharmacology mechanism of bevacizumab. Previous studies had proved bevacizumab blocks the binding of VEGF to VEGFR and reduces the level of nitric oxide that contributes to vasodilatation and causes adverse effects, such as proteinuria and hypertension [30]. In clinical practice, patients should control high blood pressure to normal levels before taking medication and routinely monitor blood pressure and urine routine tests, while taking medication. Moreover, age‐related macular degeneration using intravitreal syringes was the main reason for off‐label use, despite the lack of official approval [31, 32].

In our study, Avastin was associated with red blood cell disorders, ureteric disorders and bladder and bladder‐neck disorders. A retrospective study reported that the combination of bevacizumab, capecitabine and cyclophosphamide was associated with macrocytosis in the treatment of metastatic breast cancer; however, direct evidence linking this effect solely to Avastin remains lacking [33]. Wang et al. reported polycythaemia secondary to bevacizumab use, although the influence of disease progression and co‐administration cannot be excluded [34]. Reports on ureteric and bladder disorders related to bevacizumab are limited, but one case study did describe renal pelvic perforation following Avastin treatment [35]. These findings suggest that clinicians should monitor both renal and urinary system function when administering Avastin. Biosimilars, on the other hand, were more frequently associated with immune‐mediated renal disorders. These differences may stem from variations in manufacturing processes, excipients or formulation; nevertheless, additional studies are required to confirm this relationship. In patients with chronic kidney disease, autoimmune disorders or a history of hypersensitivity to bevacizumab, a careful assessment of treatment appropriateness is essential.

Our study, based on the FAERS database, investigated differences in AE signalling between Avastin and its biosimilars. We identified notable differences in the distribution of AEs at the SOC level, as well as in the association of specific AEs within key organ systems. Following a comprehensive analysis of these discrepancies, our findings provide meaningful implications for clinical drug selection. In practice, patient health status and available safety evidence should also be carefully weighed alongside therapeutic indications and cost considerations. Meanwhile, long‐term monitoring of manufacturing processes should be reinforced to support improved safety oversight by regulatory authorities.

### 4.3 Limitations

Despite its significance, this study has several limitations. First, FAERS‐based pharmacovigilance studies lack a standardized methodology, which may lead to variability in the quality of AE reports and potential misclassification between Avastin and its biosimilars [36, 37]. Second, FAERS is a spontaneous reporting system and substantial missing data for key variables, such as age and sex may impair statistical power and limit the generalizability of the findings. Third, specific parameters for culture, purification, filtration during monoclonal antibody production are not publicly available, making it difficult to analyse specific processes in depth. Moreover, the effects of combination therapy, patient health status and disease progression cannot be excluded, although these are often inherent characteristics of tumour patients.

## 5 Conclusion

There are slight differences in the SOC distribution between Avastin and bevacizumab biosimilars at both the HLGT and PT levels within the four key SOCs. Hypertension, proteinuria, thrombocytopenia, gastrointestinal perforation and thromboembolism remain key concerns. Red blood cell disorders, ureteric disorders and bladder and bladder‐neck disorders related to Avastin may be specific AEs and clinicians should monitor both renal function and urinary system function when administering Avastin. The observed association between biosimilars and immune‐induced renal disorders highlights the importance of assessing treatment appropriateness in patients with chronic kidney disease, autoimmune disorders or other comorbidities.

## Conflicts of Interest

The authors declare no conflicts of interest.

## Supporting information


**Table S1:** Number of PTs under Each HLGT for Avastin and Its Biosimilars in Key SOCs.

**Table S2:** Manufacturer and Formulation Details of Avastin and Its Five Biosimilars.

**Table S3:** Comparison of Antibody Quality Attributes between Avastin and Its Biosimilars.

## Data Availability

The data that support the findings of this study are openly available in FDA Adverse Event Reporting System (FAERS) at https://fis.fda.gov/extensions/FPD‐QDE‐FAERS/FPD‐QDE‐FAERS.html, reference number 2020 Q1 to 2024 Q4.
