# Supplementary material for: Comparative Safety Analysis of Avastin and Bevacizumab Biosimilars Based on Food and Drug Administration Adverse Event Reporting System
Source: Basic Clin Pharmacol Toxicol. 2025 Aug 27;137(4):e70099. doi: 10.1111/bcpt.70099 (PMC12391571; doi:10.1111/bcpt.70099)
Supplement: Supplementary file 1 — Table S1: Number of PTs under Each HLGT for Avastin and Its Biosimilars in Key SOCs. [file BCPT-137-0-s003.docx]

Supplementary Table 1 Number of PTs under Each HLGT for Avastin® and Its Biosimilars in Key SOCs

| SOC | HLGT | Number of PTs for Avastin® | Number of PTs for Biosimilars |
| --- | --- | --- | --- |
| Gastrointestinal disorders | Abdominal hernias and other abdominal wall conditions | - | 1 |
|  | Anal and rectal conditions NEC | 1 | 1 |
|  | Dental and gingival conditions | 2 | 2 |
|  | Diverticular disorders | 1 | - |
|  | Gastrointestinal conditions NEC | 7 | 4 |
|  | Gastrointestinal haemorrhages NEC | 7 | 8 |
|  | Gastrointestinal inflammatory conditions | 5 | 9 |
|  | Gastrointestinal motility and defaecation conditions | 2 | 2 |
|  | Gastrointestinal signs and symptoms | 2 | 2 |
|  | Gastrointestinal stenosis and obstruction | 7 | 7 |
|  | Gastrointestinal ulceration and perforation | 15 | 16 |
|  | Gastrointestinal vascular conditions | 3 | 6 |
|  | Oral soft tissue conditions | 2 | - |
|  | Peritoneal and retroperitoneal conditions | 3 | 5 |
|  | Tongue conditions | 1 | - |
| Vascular disorders | Aneurysms and artery dissections | 3 | - |
|  | Arteriosclerosis, stenosis, vascular insufficiency and necrosis | 2 | 2 |
|  | Coagulopathies and bleeding diatheses (excl thrombocytopenic) | 1 | - |
|  | Decreased and nonspecific blood pressure disorders and shock | 1 | 3 |
|  | Embolism and thrombosis | 10 | 8 |
|  | Lymphatic vessel disorders | 1 | - |
|  | Ocular haemorrhages and vascular disorders NEC | 1 | - |
|  | Ocular infections, irritations and inflammations | 1 | - |
|  | Retina, choroid and vitreous haemorrhages and vascular disorders | 7 | - |
|  | Spleen, lymphatic and reticuloendothelial system disorders | 1 | - |
|  | Vascular disorders NEC | 3 | 4 |
|  | Vascular haemorrhagic disorders | 2 | 1 |
|  |  |  |  |
|  | Vascular hypertensive disorders | 3 | 2 |
|  | Vascular infections and inflammations | - | 1 |
| Vascular disorders | Venous varices | 2 | 1 |
| Blood and lymphatic system disorders | Anaemias nonhaemolytic and marrow depression | 4 | 1 |
|  | Coagulopathies and bleeding diatheses (excl thrombocytopenic) | - | 1 |
|  | Haematological disorders NEC | 1 | 2 |
|  | Haemolyses and related conditions | - | 1 |
|  | Platelet disorders | 2 | 3 |
|  | Red blood cell disorders | 2 | - |
|  | Spleen, lymphatic and reticuloendothelial system disorders | 1 | 3 |
|  | White blood cell disorders | 7 | 2 |
| Renal and urinary disorders | Bladder and bladder neck disorders (excl calculi) | 1 | - |
|  | Coagulopathies and bleeding diatheses (excl thrombocytopenic) | 1 | - |
|  | Gastrointestinal conditions NEC | 1 | - |
|  | Genitourinary tract disorders NEC | 1 | 1 |
|  | Nephropathies | 3 | 12 |
|  | Renal disorders (excl nephropathies) | 9 | 4 |
|  | Ureteric disorders | 2 | - |
|  | Urinary tract signs and symptoms | 4 | 2 |
|  | Bladder and bladder neck disorders (excl calculi) | 1 | - |

Abbreviation: PT, preferred term; SOC, system organ class; HLGT, high level group term; excl, exclusion; NEC, not elsewhere classified.
