# Supplementary material for: Comparative Safety Analysis of Avastin and Bevacizumab Biosimilars Based on Food and Drug Administration Adverse Event Reporting System
Source: Basic Clin Pharmacol Toxicol. 2025 Aug 27;137(4):e70099. doi: 10.1111/bcpt.70099 (PMC12391571; doi:10.1111/bcpt.70099)
Supplement: Supplementary file 2 — Table S2: Manufacturer and Formulation Details of Avastin and Its Five Biosimilars. [file BCPT-137-0-s002.docx]

**Supplementary Table 2 Manufacturer and Formulation Details of Avastin® and Its Five Biosimilars**

| Category | Brand name | Manufacture | Formulations |
| --- | --- | --- | --- |
| Originator | Avastin® | GENENTECH | 25 mg/mL; supplied in 100 mg/4 mL and 400 mg/16 mL vials. Excipients: α, α-trehalose dihydrate, sodium phosphate (monobasic and dibasic), polysorbate 20, water for injection; pH 6.2 |
| Biosimilar | Alymsys® | AMNEAL PHARMS LLC | 25 mg/mL bevacizumab-maly;100 mg/4 mL and 400 mg/16 mL vials; Excipients: trehalose dihydrate, monobasic sodium phosphate, dibasic sodium phosphate, polysorbate 20, water for injection; pH 6.2 |
| Biosimilar | Avzivi® | BIO-THERA SOLUTIONS LTD | 25 mg/mL; 100 mg/4 mL and 400 mg/16 mL vials; Excipients: α, α-trehalose dihydrate, disodium hydrogen phosphate, sodium dihydrogen phosphate monohydrate, polysorbate 20, water for injection; pH 6.1. |
| Biosimilar | Mvasi® | AMGEN INC | 25 mg/mL ABP215; supplied in 100 mg/4 mL and 400 mg/16 mL vials; Excipients: α, α-trehalose dihydrate, sodium phosphate monobasic monohydrate, sodium phosphate dibasic anhydrous, polysorbate 20; pH 6.2 |
| Biosimilar | Vegzelma® | CELLTRION | 25 mg/mL bevacizumab-adcd;100 mg/4 mL and 400 mg/16 mL vials; Excipients: α, α-trehalose dihydrate, dibasic sodium phosphate, monobasic sodium phosphate, polysorbate 20, water for injection; pH 6.2 |
| Biosimilar | Zirabev® | PFIZER INC | 25 mg/mL bevacizumab-bvzr;100 mg/4 mL and 400 mg/16 mL vials; Excipients: sucrose, EDTA, polysorbate 80, water for injection; pH 5.5 |
