# Supplementary material for: Comparative Safety Analysis of Avastin and Bevacizumab Biosimilars Based on Food and Drug Administration Adverse Event Reporting System
Source: Basic Clin Pharmacol Toxicol. 2025 Aug 27;137(4):e70099. doi: 10.1111/bcpt.70099 (PMC12391571; doi:10.1111/bcpt.70099)
Supplement: Supplementary file 3 — Table S3: Comparison of Antibody Quality Attributes between Avastin and Its Biosimilars. [file BCPT-137-0-s001.docx]

**Supplementary Table 3 Comparison of Antibody Quality Attributes between Avastin® and Its Biosimilars**

| Brand name | Quality attributes of antibody (Biosimilars vs. Avastin®) | | | | | | |
| --- | --- | --- | --- | --- | --- | --- | --- |
|  | Primary Structure | N-linked Glycosylation | Higher-Order Structure | Charge Variants | Product-Related Variants | Bioactivity | Drug Product Attributes |
| Alymsys® | Highly similar, minor substitutions Cys→Tyr | Increased afucosylation (4.29-6.00% vs. 1.72-6.08%), increased galactosylation, increased sialylation (0.3-0.7% vs. 0.0-0.3%) | Higher free thiols (0.008791-0.010541 vs. 0.006808-0.009486 mol/mol) | Wider acidic species range (18.3-25.4% vs. 20.8-23.9%) | Higher monomer purity, lower HMW species (0.7-2.2% vs. 2.1-3.2%), higher HHL | FcγRIIIa binding (158V and F) outliers | Early-batch higher protein concentration (25.4-27.5 vs. 23.2-25.4 mg/mL) |
| Avzivi® | Highly similar | Increased galactosylation (19.0-28.9% vs. 3.09-18.51%), increased afucosylation (2.1-5.1% vs. 1.22-2.78%), increased sialic acid (0.6-1.4% vs.0-0.3%) | - | Wider acidic species range (12.5-27.5% vs. 22.2-31.9%) | Higher monomer purity, lower Non-glycosylated HC (0.36-0.42% vs. 1.2-2.2%) | FcγRIIIa 158V binding outliers | Slightly higher protein concentration (24.2-26.55 vs. 23.1-26.0 mg/mL) |
| Mvasi® | Highly Similar | Higher galactosylation (10.0-21.8% vs. 7.8-21.2%), higher afucosylation (1.6-3.8% vs. 1.9-2.6%) | - | Lower level of acidic peak (24.2-29.5% vs. 25.7-30.7%) | Higher monomer purity, less aggregates (2.4-3.3% vs. 2.3-3.4%) | Slight FcγRIIIa and FcγRIIIb binding difference | - |
| Vegzelma® | Highly Similar | Lower glycation (1.7-2.2% vs. 2.9-4%), higher fucosylated G0F glycans (79.94-84.74% vs. 76.23-80.24%) | - | Lower acidic variants (13.6-15.7% vs. 19.40-21.32%), higher basic variants (7.4-12.3% vs. 3.29-7.09%) | Higher monomer purity, lower HMW species (0.7-2.2% vs. 1.85-2.39%) | - | Slightly higher protein concentration (24.9-26.3 vs. 25.25-25.97 mg/mL) |
| Zirabev® | Highly Similar | Higher high mannose (1.3-3.1% vs. 0.5-1.1%), higher Afucosylation (2.3-4.6% vs. 2.4-3.3%), higher Galactosylation (13.0-22.1% vs. 6.5-18.9%) | - | Higher basic peaks (6.0-17.4% vs. 3.6-6.5%) due to C-terminal lysine residues | Higher monomer purity, Fewer fragments (0.7-1.0% vs. 1.7-3.6%), higher intact IgG (96.6-98.0 vs.95.7-97.7) | Slightly higher binding affinity to VEGFA 206 (no clinical impact) | Slightly lower protein concentration (23.6-25.0 mg/mL vs. 24.3-26.3 mg/mL) |

Abbreviation: Cys, cysteine; Tyr, tyrosine; HMW, high molecular weight ;HHL, heavy-heavy-light; FcγR, Fc gamma receptor; HC, heavy chain; G0F, G0F-type N-glycans; VEGFA, vascular endothelial growth factor a.

Note: Observed quality attribute differences reflect minor clinically inactive component variations. Per approval documentation, these differences do not prevent the biosimilar from being deemed highly similar to Avastin® (reference product) nor impede the scientific bridge's analytical component.
